# Tumors of the Testicle, with Remarks on a Case of Hæmatocele

**Published:** 1849-09

**Authors:** A. H. Grimshaw

**Affiliations:** Wilmington, Del., late Physician to the “Orphans’ Shelter,” Philadelphia


*Tumors of the Testicle, with remarks on a case of Hæmatocele;* By A. H. Grimshaw, of Wilmington, Del., late Physician to the "Orphans' Shelter," Philadelphia.

Much difficulty exists in forming a diagnosis in tumors of the testicle. Hydrocele has been mistaken for hernia; a case is reported in which the operator went so far as to lay open the sac; only discovering his error when the fluid followed the knife.

Hæmatocele and hydrocele may be confounded, also hæmatocele and medullary fungus; hæmatocele and hernia, might be more easily confounded than the latter and hydrocele.

I present to you a short history of a case, lately coming under my notice.

J. W——, paper maker, temperate, æt. 40, consulted me, about the 1st inst., concerning a tumor of the right testicle, or, as he expressed it, "a growing of one testicle into the other." Latterly he had felt some pain in the groin, and the fear of hernia hastened his desire for relief. The swelling was elastic, certainly more so than fluctuating; more solid than hydrocele, with less corrugation of scrotum; no transparency.

I suggested tapping, calling it hydrocele, in which decision my friend, Dr. Goddard, agreed. It was my opinion, however, that the fluid was contained within the tunica albuginea, and that this occasioned the deceptive feeling of solidity. At least six years had elapsed since first noticing, and for some years no increase had taken place ; until lately no pain had been present. A suspensory bandage relieved the pain entirely, thereby proving the non-malignancy of the tumor.

On the 8th inst. I tapped, intending to use an injection of tinct. iodine and water. At least a pint of bloody, homogeneous fluid flowed out. The sac was thickened ; did not contract ; there was also circocele and enlargement of the vas deferens. The testicle lay in front and at the bottom of the tumor.

I deferred the use of the injection, believing that a better cure would be made by allowing the sac time to contract.

It will be perceived, that we were right as to the fact of fluid being present, but wrong as to the precise nature of the disease. Under the circumstances, I think I was right in hesitating to inject an irritating fluid, since I found, in place of hydrocele, hæmatocele. The latter is generally laid down in books as occurring suddenly, and after tapping for hydrocele. Pott, however, describes an effusion of blood within " the tunica albuginea, or proper coat of the testicle, and if the quantity be considerable, it will afford or produce fluctuation to the hand of the examiner, very like to that of an hydrocele of the tunica-vaginalis." He further says, that " if this be mistaken for simple hydrocele, and an opening be made, the discharge will be blood ; not fluid, or very thin ; not like to blood circulating in its proper vessels ; but dark and dusky in color, and nearly of the consistence of chocolate." According to this author, extirpation of the testicle is the only remedy.

The most delicate touch cannot reveal to us whether we have, in the tumor before us, water or a bloody fluid. Neither can we base our diagnosis on the transparency, or the reverse No great injury would be caused by puncturing even a sarcocele, or like solid tumor of the testicle, except that " dry tapping," as in some other situations, seldom redounds to the credit of the operator.

I have remarked above, that I believed this fluid was water ; but that it was contained within the tunica albuginea. In an ordinary hydrocele, we would only have as coverings of the fluid, integuments, cellular tissue, dartos and tunica vaginalis; none of these separately, nor all conjoined, possessing a structure such as to produce that "deceptive feeling of fluctuation." The tunica albuginea, however, is "dense strong, and fibrous, resembling in structure the tunica sclerotica.

In fact, I might have compared the impression of this tumor upon the hand, to that made by an eye from a large animal, such as the ox.

Chelius speaks of medullary fungus, and of "the absence of all hardness and irregularity, and the very delusive feeling of fluctuation, and the very indistinct pain;" these symptoms were present, but the pain only for a month or two; its growth was slow; fungus is rapid in growth.

Collections of fluid, under fibrous membranes, are especially apt to be mistaken for encysted tumors, requiring the use of the knife. Within my knowledge, a surgeon operated with success upon an abscess under the fascia lata of the thigh, hoping, no doubt, the evidence of his skill would grace his pathological cabinet. It is this doubt, this uncertainty, the memory of opinions given, and not borne out by the event of the case, that causes our patients "rather to bear the ills they have," than trust a knife, which may not eradicate the cause of their suffering.

At this moment the remembrance of a case, of what was supposed, before operation, to be enlarged testis, requiring excision, comes to my mind. ——, married, &c., came to the Philadelphia Dispensary in 1844, and was directed to the surgeon then on duty, who kindly obtained admission for him to the Pennsylvania Hospital. It was there agreed to extirpate—the patient was placed on the table, an incision made, when, to the astonishment of all present, a *bony sac*, containing fluid, and a perfectly healthy testis, were found.

In this case there was hardness and pain and no transparency: the history of the case threw no light upon it. Sir Charles Bell mentions, that in old hydroceles, "the tunica vaginalis becomes much thickened and even ossified." So we find another landmark, in making our diagnosis, is uprooted. We may find this bony case, very irregular, probably knobby, giving the idea of a sarcocele, which can be readily cured by appropriate medical treatment.

My assertion, that the bag truss having relieved the dragging and pain, proved non-malignancy, may be called in question. It is said "sarcomatous degeneration of the testicle" does not produce, for a long time, more than a dragging and slight pain, which is removed by a bag truss.

Again, we may confound cystic swelling of the testicle with hydrocele, and much more easily, with hæmatocele.

With hernia, hæmatocele might be, even by a careful surgeon, confounded. A patient has had a pain in the groin; he also says, that for some days he has had no evacuation from the bowels. Some men are thrown into paroxysms of pain by the mere mention of an examination; the approach of a hand causes them to groan; while others, actually in more danger, will allow you to handle them very roughly without an exclamation.

Suppose one of these irritable subjects is attacked with cholic, calls a physician, who, being a careful man, and having his attention drawn to hernia by the complaint of cholic, enquires whether his patient has a rupture; being told there is a tumor in the pubic region, he examines: in all probability, having suspected hernia from the first, he jumps at a conclusion, endeavors to reduce by taxis, &c., and, as in cases on record, his astonishment is only equalled by his chagrin when his error is discovered.

It remains to be proved whether Potts's assertion is correct, as to the necessity of extirpation.

*May* 15*th*, 1849.

